# Pre-shaping double-lumen endotracheal tubes based on video laryngoscope blade curvature enhances first-attempt intubation success rate in patients undergoing thoracoscopic surgery: a randomized controlled trial

**DOI:** 10.3389/fmed.2025.1700773

**Published:** 2026-01-08

**Authors:** Yanling Yang, Qian Liu, Fengfeng Xiao, Lei Yang, Hui Liu, Yuchan Wang, Min Liu, Jie Tang, Qi Wang, Jie Lv, Hongyan Zhang, Liu Xu, Wei Wang

**Affiliations:** 1 Department of Anesthesiology, Wenjiang Hospital of Sichuan Provincial People's Hospital, Chengdu Wenjiang District People's Hospital, Chengdu, China; 2 Department of Anesthesiology, The Affiliated Jiangning Hospital of Nanjing Medical University, Nangjing, China; 3 Department of Research and Education, Wenjiang Hospital of Sichuan Provincial People's Hospital, Chengdu Wenjiang District People's Hospital, Chengdu, China

**Keywords:** double-lumen endotracheal tube, video laryngoscopy, tracheal intubation, tube shaping, thoracoscopic procedures

## Abstract

**Background:**

The widespread adoption of video laryngoscopes (VLs) in double-lumen endotracheal tube (DLT) intubation has not resolved controversies regarding their efficacy in improving first-attempt success rates. This study aimed to evaluate the safety and efficacy of pre-shaping DLTs according to the VL blade curvature for tracheal intubation in patients undergoing thoracoscopic surgery.

**Methods:**

A total of 90 patients scheduled for elective thoracoscopic surgery under general anesthesia with left-sided DLT intubation were enrolled. The patients were aged ≥18 years and had an American Society of Anesthesiologists (ASA) physical status I or II. All patients were divided into two groups using a random number table method: a video laryngoscope blade curvature-based pre-shaping group (Group P, *n* = 45) and a traditional empirical shaping group (Group T, *n* = 45). All patients underwent total intravenous anesthesia and were intubated under VL. The primary outcome was the first-attempt intubation success rate. Secondary outcomes included tube malposition rate, time for DLT passage through the glottis, total intubation time, mean arterial pressure (MAP), and heart rate (HR) at baseline (pre-induction), post-induction, during intubation, and 5 min post-intubation. Complications related to intubation within 48 h after surgery, such as pharyngolaryngeal pain, hoarseness, and lip and dental injuries, were also documented.

**Results:**

Compared to Group T, Group P demonstrated a significantly higher first-attempt intubation success rate (91.1% vs. 72.7%, *p* < 0.01), shorter time for tube passage through the glottis (14.2 ± 3.1 vs. 29.5 ± 4.8 s, *p* < 0.01), and reduced total intubation time (58.3 ± 10.2 vs. 82.6 ± 12.4 s, *p* < 0.01). During intubation, Group P exhibited significantly lower MAP and HR compared to Group T (*p* < 0.05). Furthermore, the incidence of postoperative pharyngolaryngeal pain (13.3% vs. 31.8%, *p* < 0.01) and lip injuries (6.7% vs. 18.2%, *p* < 0.01) within 48 h was markedly lower in Group P.

**Conclusion:**

Pre-shaping DLTs based on VL blade curvature improves the first-attempt intubation success rate while minimizing intubation-related trauma in thoracoscopic surgery.

**Clinical trial registration:**

https://www.chictr.org.cn/bin/home, identifier ChiCTR2400080906.

## Introduction

The double-lumen endotracheal tube (DLT) is the most widely used device for one-lung ventilation in thoracic surgery (1). Compared to single-lumen tubes, DLTs feature an irregular oval cross-section, larger diameter, and greater rigidity, which significantly increase intubation difficulty, prolong procedure duration, and increase failure rates (2). Additionally, the inherent angulation between the bronchial lumen and tracheal orifices, designed to facilitate bronchial entry, amplifies insertion resistance, predisposing patients to airway trauma and higher incidences of postoperative pharyngolaryngeal pain, lip injuries, and hoarseness compared to single-lumen intubation (3). Studies have suggested that modifying DLT shaping (4), thermal softening (5), or rotational techniques (6) may mitigate these complications.

Video laryngoscopy (VL) enhances glottic visualization and improves the first-attempt intubation success rate, making it invaluable for difficult airway management (7–9). However, its efficacy in DLT intubation remains controversial. Risse et al. (10) found that VL improved glottic exposure without increasing first-attempt success compared to Macintosh laryngoscopy. Conversely, Kim et al. (11) reported a higher success rate with VL, but prolonged intubation times and increased tube misplacement. Karczewska et al. (12) observed shorter intubation times with VL but no success rate improvement, whereas a retrospective study by Kına et al. (13) demonstrated reduced DLT misplacement and complications with VL. These discrepancies may arise from inconsistent DLT shaping protocols: insufficient angulation prevents the tip from reaching the glottis, whereas excessive curvature causes tip deflection or forms an angle with the glottis, making it difficult for the DLT to enter the trachea (4). Despite the DLT’s preformed design, traditional empirical shaping lacks standardization, resulting in a variable first-attempt success rate (53.7–83.3%) under VL guidance (12, 14, 15). This study introduces a novel VL blade curvature-based pre-shaping method for DLTs, demonstrating superior clinical outcomes to provide a practical reference for DLT intubation in clinical settings.

## Methods

### Ethics and registration

This study followed the Consolidated Standards of Reporting Trials (CONSORT) recommendations (16). Ethical approval for this study (No. 2024-003) was provided by the Ethics Committee of the Chengdu Wenjiang District People’s Hospital. The study was registered at the Chinese Clinical Trial Registry (No. ChiCTR2400080906) on 18 February 2024. All participants were informed of the proposal and provided written informed consent. All study procedures were conducted in accordance with good clinical practice guidelines and adhered to the tenets of the Declaration of Helsinki.

### Participants

Patients scheduled for elective thoracoscopic surgery under general anesthesia with left-sided DLT intubation at the Chengdu Wenjiang District People’s Hospital between 1 March 2024 and 31 January 2025 were enrolled in the study. Inclusion criteria were as follows: age ≥18 years, BMI 18.5–28 kg/m^2^, ASA physical status I or II, regardless of sex. Exclusion criteria were as follows: (1) difficult airway (17) (interincisor distance <3 cm, thyromental distance < 6 cm, modified Mallampati classification >III, or significant head/neck mobility limitation); (2) contraindications to DLT intubation; (3) severe dysfunction of major organs (cardiac, cerebral, or pulmonary); (4) alterations in airway management strategy; (5) deviations from predefined tube shaping methods; and (6) intraoperative DLT size changes.

### Sample size calculation

Based on previous studies (12, 14, 15), the first-attempt intubation success rate using empirically shaped DLTs under VL was approximately 70%. Our pilot study (10 participants in each group) demonstrated that the novel VL blade curvature-based pre-shaping method achieved a success rate of 90%. With a significance level (*α*) of 0.05 and power (1−*β*) of 0.8, the calculated minimum sample size was 78 participants. Accounting for a 10% anticipated attrition rate, the final study cohort was expanded to 90 patients, with 45 patients allocated to each group.

### Randomization and blinding

Participants were randomly allocated to one of the two groups. A randomization sequence was generated using SPSS 26.0 software (SPSS Inc., Chicago, IL, United States), and an independent statistician uninvolved in the trial prepared 90 sequentially numbered, sealed opaque envelopes. Both the participants and outcome assessors were blinded to the group assignments. To ensure allocation concealment, group assignments were secured in sealed opaque envelopes until intervention initiation. An anesthesiologist who was not involved in the study opened the envelopes immediately before intubation to reveal the group allocation.

### Interventions

**Figure 1:**
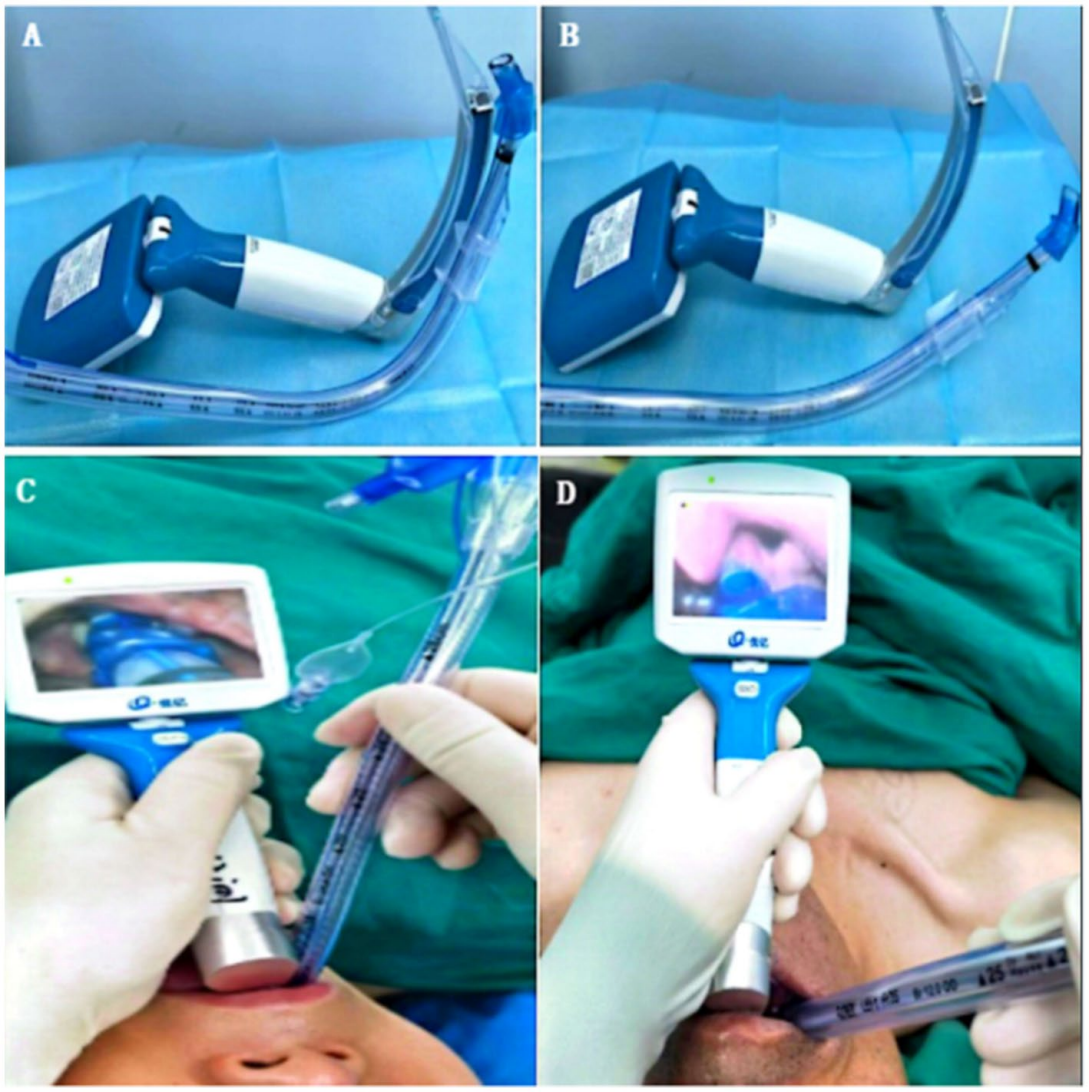
Pre-shaped DLTs and intubation procedure diagram. **(A)** DLTs were pre-shaped to match the curvature of the VL blade in Group P. **(B)** Empirical shaping was performed in Group T. **(C)** Diagram of the DLT intubation procedure in Group P. **(D)** Diagram of the DLT intubation procedure in Group T. DLT, double-lumen endotracheal tube; VL, video laryngoscope.

All patients fasted for 12 h and abstained from fluids for 6 h preoperatively, with no premedication administered. Upon entering the operating room, peripheral intravenous access was established. Standard monitoring included electrocardiography (ECG), heart rate (HR), peripheral oxygen saturation (SpO₂), non-invasive blood pressure (NIBP), end-tidal carbon dioxide partial pressure (P_ET_CO₂), and bispectral index (BIS). Radial artery cannulation under local anesthesia was performed for continuous invasive arterial pressure monitoring. Anesthesia was induced with preoxygenation (8 L/min for 5 min) followed by sequential intravenous administration of midazolam (0.05 mg/kg), etomidate (0.2 mg/kg), rocuronium (0.6 mg/kg), and fentanyl (4 μg/kg) (18–21). Tracheal intubation was performed after achieving complete neuromuscular blockade using standardized DLT (125,037, Henan Pucan Medical Equipment Co., Ltd., China) sizes (Fr = 35# for males, Fr = 32# for females; intraoperative size changes led to exclusion). DLTs were pre-shaped to match the curvature of the VL (TD-C-IV, Zhejiang UE Medical Co., Ltd., China) blade in Group P (Figure 1A), while empirical shaping was performed in Group T (Figure 1B). Both groups used identical VL-guided intubation techniques. The VL blade was inserted via the right oral commissure, displacing the tongue posteriorly to expose the glottis, and the DLT (with the tip oriented upward) was advanced until the bronchial cuff passed through the vocal cords (Figures 1C,D). After the bronchial cuff passed through the glottis, the tube was rotated counterclockwise by 90° (1, 22). Upon encountering resistance, a 90-degree clockwise rotation was applied before stylet removal by an assistant. After fiberoptic bronchoscopic confirmation of proper DLT positioning, mechanical ventilation was initiated (Dräger Primus^®^, Shanghai Dräger Medical Equipment Co., Ltd., Germany) in intermittent positive-pressure ventilation (IPPV) mode, with dual-lung ventilation parameters set at tidal volume (VT) 8–10 mL/kg ideal body weight, inspiratory-to-expiratory (I:E) ratio 1:2, and fraction of inspired oxygen (FiO₂) 60%, transitioning to one-lung ventilation with VT 6–8 mL/kg, I:E ratio 1:2, and FiO₂ 70% (23, 24). During dual-lung and one-lung ventilation, the respiratory rate (RR) was set to maintain an end-tidal carbon dioxide partial pressure (P_ET_CO₂) of 35–45 mmHg (25, 26). General anesthesia was maintained through continuous intravenous infusions of propofol (4–8 mg/kg/h), remifentanil (0.1–0.3 μg/kg/min), and cisatracurium (0.1–0.2 mg/kg/h), titrated to achieve BIS values of 45–60. The neuromuscular blockade was discontinued 30 min prior to surgical conclusion by stopping cisatracurium, while propofol and remifentanil infusions were continued until skin closure. Postoperative analgesia was initiated with an intravenous injection of sufentanil (0.1 μg/kg) for a loading dose, followed by a patient-controlled intravenous analgesia (PCIA) pump containing sufentanil 1.5 μg/kg and tropisetron 0.05 mg/kg diluted to 100 mL with normal saline, programmed with a 2 mL/h basal infusion, 1 mL bolus doses, and a 15 min lockout interval. Postoperatively, the DLT was retracted to the trachea under fiberoptic guidance, and extubation in the postanesthesia care unit (PACU) required a Steward recovery score >4 (27), with criteria including consciousness, muscle strength, respiratory rate >12 breaths/min, tidal volume >5 mL/kg, and intact reflexes. All intubations and bronchoscopic verifications were performed by a single associate chief anesthesiologist with >10 years of experience in DLT intubation, assisted by a dedicated nurse anesthetist. Surgical procedures were conducted by a consistent thoracic surgical team.

### Main outcome measures

The primary outcome was the first-attempt intubation success rate, defined as successful advancement of the DLT through the glottis into the left main bronchus on the initial attempt. Secondary outcomes included (1) DLT malposition rate (incidence of right main bronchial placement); (2) DLT passing glottis time (interval from intubation initiation to bronchial cuff passage through the glottis); (3) total intubation time (from intubation initiation to fiberoptic bronchoscopic confirmation of correct positioning); (4) MAP and HR at pre-induction, post-induction, during intubation, and 5 min post-intubation; (5) emergence time (surgery end to eye-opening response to verbal commands); (6) extubation time (surgery end to tracheal tube removal); and (7) complications related to intubation within 48 h after surgery (pharyngolaryngeal pain, hoarseness, lip and dental injuries).

### Statistical analysis

Data analysis was performed using SPSS 26.0 (SPSS Inc., Chicago, IL, United States). Normality of continuous variables was assessed using the Shapiro–Wilk test. Normally distributed data are expressed as mean ± standard deviation (*x-* ± s) and were compared between groups using an independent-samples *t*-test. Non-normally distributed data were reported as medians with interquartile ranges [M (IQR)] and analyzed using the Mann–Whitney U test. Categorical variables are presented as numbers (%) and were evaluated using the χ^2^-test or Fisher’s exact test. A two-tailed *p*-value of <0.05 was considered statistically significant.

## Results

### Participant enrollment

**Figure 2:**
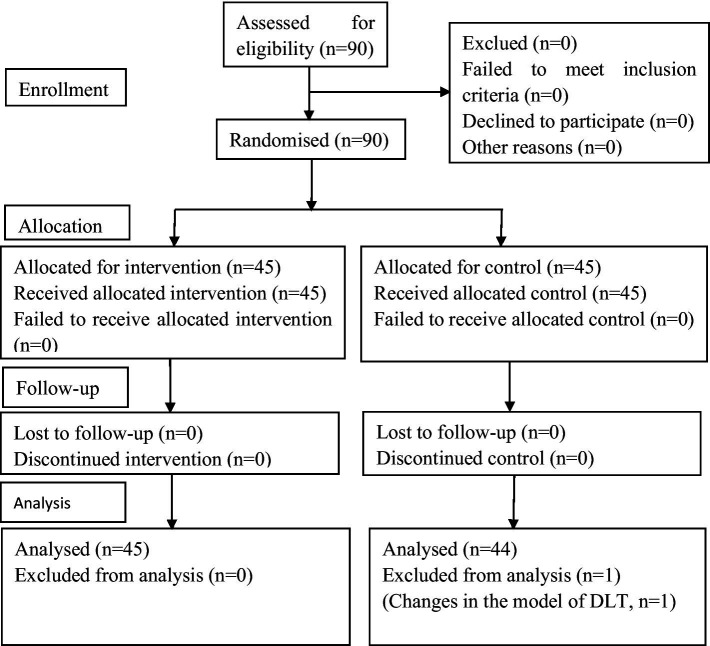
CONSORT flow diagram. In this study, 90 patients were initially enrolled, and 1 patient was excluded. In total, data from 45 patients in Group P and 44 patients in Group T were analyzed. CONSORT, Consolidated Standards of Reporting Trials.

Initially screened, 90 patients were randomized, with one male patient in Group T excluded due to an intraoperative DLT size change (Fr = 32#). Consequently, 45 cases in Group P and 44 cases in Group T were included in the final analysis (Figure 2).

### Demographic data and patient characteristics

No statistically significant differences were observed between the groups in age, sex, BMI, ASA classification, surgical duration, awakening time, and extubation time (Table 1).

**Table 1:** Comparison of baseline characteristics in the two groups.

Characteristic	Group P (*n* = 45)	Group T (*n* = 44)	*p-*value
Age (years old)	58.7 ± 11.6	54.8 ± 14.0	0.153
Gender, Male/Female [*n* (%)]	22/23 (48.9%/51.1%)	24/20 (54.5%/45.5%)	0.673
BMI (kg/m^2^)	23.5 ± 3.2	24.4 ± 4.2	0.248
ASA class, II/III [*n* (%)]	31/12 (68.9%/31.1%)	34/10 (77.3%/22.7%)	0.357
Duration of operation (min)	121.3 ± 53.9	121.4 ± 48.6	0.993
Awakening time (min)	9.1 ± 1.2	9.0 ± 1.3	0.822
Time to extubation (min)	25.3 ± 7.6	23.6 ± 5.2	0.638

Data are presented as the mean ± SD and case numbers (percentage). No significant differences were observed between the two groups (*P* ≥ 0.05); mutual comparison was analyzed using an independent-samples *t*-test and χ^2^-test; BMI, body mass index; ASA, American Society of Anesthesiologists.

### Intubation outcomes

Compared with Group T, Group P exhibited a significantly higher first-attempt intubation success rate (*p* < 0.01), shorter glottis passage time of the DLT (*p* < 0.01), and reduced total intubation time (*p* < 0.01). No difference was found in the DLT malposition rate between the two groups (Table 2).

**Table 2:** Indicators of intubation in the two groups.

Group	First-attempt intubation success rate [*n*(%)]	DLT malposition rate [*n*(%)]	Time of DLT passage through the glottis (s)	Total intubation time (s)
Group P (*n* = 45)	41 (91.1)^a^	3 (6.7)	14.2 ± 3.1^a^	58.3 ± 10.2^a^
Group T (*n* = 44)	32 (72.7)	4 (9.1)	29.5 ± 4.8	82.6 ± 12.4
*p-*value	0.003	0.858	0.008	0.006

Data are presented as mean ± SD and case numbers (percentage). Mutual comparisons were analyzed using an independent samples *t*-test and χ^2^-test; compared with group T, ^a^*P* < 0.01. DLT, double-lumen endotracheal tube.

### Hemodynamic profiles

Group P demonstrated significantly lower MAP and HR during intubation compared to Group T (*p* < 0.01). No significant hemodynamic differences were observed at pre-induction, post-induction, or 5 min post-intubation between the two groups (Table 3).

**Table 3:** Different points of hemodynamics in the two groups.

Time point	(mmHg)		*p-*value	HR (beat/min)		*p-*value
	Group P (*n* = 45)	Group T (*n* = 44)		Group P (*n* = 45)	Group T (*n* = 44)	
Pre-induction	129.6 ± 13.1	133.2 ± 10.9	0.368	66.3 ± 5.7	64.9 ± 6.5	0.557
Post-induction	117.6 ± 9.5	115.8 ± 8.4	0.802	61.7 ± 5.2	62.8 ± 5.9	0.222
During intubation	122.3 ± 10.7	145.6 ± 11.2	0.009	67.4 ± 7.1	78.8 ± 8.3	0.012
Five minutes post-intubation	113.8 ± 9.1	120.1 ± 10.6	0.394	62.6 ± 4.6	65.2 ± 5.6	0.427

Data are presented as mean ± SD. Mutual comparisons were analyzed using an independent-sample *t*-test; compared with group T, ^a^*P* < 0.05. MAP, mean arterial pressure; HR, heart rate.

### Complications of intubation

Compared with Group T, the incidence of postoperative pharyngolaryngeal pain and lip injuries within 48 h was significantly lower in Group P (*p* < 0.01). No differences were detected in hoarseness or dental injuries between the two groups (Table 4).

**Table 4:** Complications of intubation in the two groups.

Group	Pharyngolaryngeal pain	Hoarseness	Lip injury	Dental injury
Group P (*n* = 45)	6 (13.3)^a^	3 (6.7)	3 (6.7)^a^	0 (0)
Group T (*n* = 44)	14 (31.8)	4 (9.1)	8 (18.2)	0 (0)
*P-*value	0.002	0.788	0.006	

Data are presented as case numbers (percentage). Mutual comparisons were analyzed using the χ^2^-test; compared with group T, ^a^*P* < 0.01.

## Discussion

The widespread adoption of VL in DLT intubation has not resolved controversies regarding its efficacy in improving the first-attempt success rate (10–13). This study demonstrates that pre-shaping DLTs according to VL blade curvature significantly enhances the first-attempt success rate, reduces intubation time, and minimizes tissue trauma, offering a potential solution to these ongoing discussions.

DLT intubation involves four critical steps: glottis exposure, bronchial tip alignment with the glottis, tube rotation, and advancement into the target bronchus (28). Among these steps, precise bronchial tip delivery through the glottis remains the most technically demanding aspect of VL (29). Suboptimal tube shaping directly compromises the success rate: insufficient curvature impedes glottis entry, while excessive angulation causes tip deflection or impaction against the glottis (30). Previous studies comparing VL and conventional laryngoscopy for DLT intubation reported inconsistent outcomes, likely due to heterogeneous shaping protocols (4). For instance, Risse et al. (10) found that GlideScope VL improved glottis visualization without increasing first-attempt success, which is potentially attributable to mismatched tube shaping (high-angle GlideScope vs. flat-angled GlideSite^®^ stylet). This underscores the importance of harmonizing VL blade geometry with DLT shaping, as VL eliminates the need for pharyngeal axis alignment yet requires tube curvature optimization (31). Traditional empirical shaping fails to achieve optimal blade-tube congruence, often resulting in restricted visual fields and iatrogenic injuries (30). Our protocol, which mimics VL blade curvature during DLT pre-shaping, aligns biomechanically with the glottis anatomy, maximizing intubation space and resistance (32).

The results of this study demonstrated that the first-attempt success rate of traditional empiric pre-shaped DLT intubation was 72.7%, which aligned with the findings (73%) reported by Chastel et al. (14). In contrast, VL blade curvature-based pre-shaping DLT intubation showed significantly improved first-attempt success (91.1%), potentially attributable to enhanced glottic space visualization, optimized visual field, and facilitated DLT passage through the vocal cords (33). No significant difference in the tube malposition rate was observed between the two groups (6.7% vs. 9.1%), consistent with the 9.7% malposition rate reported by Guan et al. (34) for conventional VL-guided DLT intubation. This similarity may be related to the inherent curvature of the left-sided DLTs and rotational maneuvers during intubation. Cameron et al. (29) demonstrated that a 180-degree rotation of DLTs reduced the mean intubation angle between the DLT and proximal trachea, thereby improving the first-attempt success rate. From an anatomical perspective, the angle between the left main bronchus and proximal trachea exhibits interindividual variability. Zhou et al. (6) revealed that preoperative CT-based individualized DLT rotation according to left main bronchial angulation improved first-attempt success and reduced malposition rate in adult patients undergoing elective thoracic surgery. This suggests that VL blade curvature-based pre-shaping suggested in our study likely enhanced first-attempt success by facilitating DLT passage through the glottis, thereby shortening the glottic transit time and consequently reducing the total intubation duration.

Compared with single-lumen endotracheal tubes, DLTs exhibit longer lengths, larger diameters, and technically more challenging intubation due to the requirement for both tracheal and bronchial lumens to pass through the glottis, thus rendering the procedure more complex and imposing greater mechanical trauma on pharyngolaryngeal tissues (35, 36). Postoperative complications such as pharyngeal pain, hoarseness, and lip and dental injuries secondary to tube compression are the most frequently reported tissue injuries following DLT intubation (37). Multiple studies have demonstrated that improper shaping of DLTs significantly increases the number of intubation attempts (38), thereby elevating the risks of hemodynamic fluctuations and postoperative complications, including pharyngolaryngeal pain (28.3–43.5%), lip injuries (15.9–35.6%), and hoarseness (6.2–19.8%) (39–41). In this study, the incidence of intubation-related complications within 48 h postoperatively in the traditional empirical DLT shaping group was comparable to these findings, with rates of pharyngolaryngeal pain, lip injuries, and hoarseness reported as 31.8, 18.2, and 9.1%, respectively. In contrast, VL blade curvature-based pre-shaping DLTs tailored to the curvature of the laryngoscope blade demonstrated significantly reduced incidences of pharyngolaryngeal pain and lip injuries (13.3 and 6.7%, respectively). This highlighted that the VL blade curvature-based pre-shaping DLT minimized repetitive tube movement in the pharyngolaryngeal region and alleviated mechanical compression on the lips (42). Wang et al. (37) reported a 0.2–12% incidence of dental injury during DLT intubation, whereas no dental injuries were observed in either group of this study. This discrepancy may be attributed to the exclusion of patients with difficult airways during the initial screening, which relatively reduced the number of intubation attempts. Furthermore, the attenuated hemodynamic fluctuations observed during intubation further validated the reduced mechanical irritation associated with this pre-shaped technique.

There are several limitations in this study. First, intubator blinding was not feasible because of the nature of the intervention. However, all intubation procedures were performed by a single experienced associate chief anesthesiologist specializing in DLT placement to minimize operator-dependent variability. Second, as postoperative sore throat was a prespecified secondary outcome, no further pain scoring or stratified analysis (e.g., by intubation duration or cuff pressure) was conducted, potentially limiting the granularity of complication assessments. Finally, this study was a single-center randomized controlled trial with a small sample size, and future multi-center, large-scale studies are warranted to validate these findings.

## Conclusion

In patients undergoing thoracoscopic surgery, VL blade curvature-based DLT pre-shaping improves the first-pass intubation success rate, reduces procedural time, and decreases pharyngolaryngeal and lip injuries. This protocol addresses the critical limitations of empirical shaping and warrants integration into thoracic anesthesia practice.

## Data Availability

The raw data supporting the conclusions of this article will be made available by the authors, without undue reservation.
